# Understanding Patient and Physiotherapist Requirements for a Personalized Automated Smartphone Telemonitored App for Posttotal Knee Arthroplasty Rehabilitation: Qualitative Study

**DOI:** 10.2196/59688

**Published:** 2025-04-14

**Authors:** Eleanor Shuxian Chew, Aileen Eugenia Scully, Samanth Shi-Man Koh, Ee-Lin Woon, Juanita Krysten Miao-Shi Low, Yu-Heng Kwan, John Wei-Ming Tan, Yong-Hao Pua, Celia Ia-Choo Tan, Luke Jonathan Haseler

**Affiliations:** 1 Department of Physiotherapy, Singapore General Hospital, Singapore, Singapore; 2 Health and Social Sciences Cluster, Singapore Institute of Technology, Singapore, Singapore; 3 Programme in Health Services and System Research, Duke-NUS Medical School, Singapore, Singapore; 4 Medicine Academic Programme, Duke-NUS Graduate Medical School, Singapore, Singapore; 5 Curtin School of Allied Health, Curtin University, Perth, Australia

**Keywords:** knee replacement, knee arthroplasty, mobile, application, interview, telemonitored, smartphone rehabilitation, mobile phone

## Abstract

**Background:**

Total knee arthroplasty (TKA) is a cost-effective surgical intervention for painful knee osteoarthritis in older adults, but postsurgery rehabilitation access is limited. Telerehabilitation offers a solution, but existing models require significant therapist involvement and a costly setup. A personalized smartphone-based automated program could be a cost-effective alternative.

**Objective:**

This study aimed to understand the requirements of both patients and physiotherapists in developing an automated telemonitored rehabilitation smartphone app for individuals undergoing TKA. To ensure uptake and long-term sustainability, this study adopted a person-based approach.

**Methods:**

A multistakeholder qualitative study of user needs was conducted. Physiotherapists and patients who underwent TKA were recruited via purposive sampling. Individual in-depth, hour-long interviews were conducted via Zoom by an experienced, trained female interviewer with a Master of Arts in Sociology. Data were audio-recorded and transcribed by the same interviewer. Two reviewers (ESC and SSK) independently analyzed the data using thematic analysis, with data triangulation achieved through cross-checking of data sources by 3 reviewers (ESC, SSK, and AES). Interviews were conducted to data saturation.

**Results:**

Six patients and 4 physiotherapists participated. For the patient interface, patients emphasized ease of use and specified features like a search function and multilingual options. For the physiotherapist interface, physiotherapists stated ease of accessing patient data and outcome measures for effective monitoring as important. Both patients and physiotherapists highlighted the need for timely, condition-specific information, supplemented by visual aids to support exercises, pain management, and recovery goals. They also stressed the significance of progress tracking, feedback, and the ability to access health care professionals for reassurance. Motivational features, including reminders, prompts, and exercise logs, were recommended to improve adherence. Both groups similarly identified the need for initial training to ensure confident use of the app.

**Conclusion:**

This study provided insights into the requirements of potential end users of a smartphone app for automated telemonitored rehabilitation following TKA. This is useful for steering the development of a user-centric smartphone app.

## Introduction

Total knee arthroplasty (TKA) is a cost-effective surgical intervention for managing painful knee osteoarthritis [1,2]. With an aging global population and rising obesity rates, TKA demand is projected to increase significantly [3]. In Singapore, all patients discharged post-TKA are referred to hospital-based outpatient physiotherapy, which improves physical function [4-6], but limited access, high costs, and logistical challenges often hinder adherence [7]. This highlights the urgent need for accessible, cost-effective physiotherapy alternatives [8,9].

A systematic review conducted by Jansson et al [10] in 2020 found that telerehabilitation for lower limb joint replacements achieved outcomes comparable to in-person physiotherapy but given the high manpower and equipment requirements, it was only cost-effective mainly for those living over 30 km from care centers [9]. In contrast, a telemonitored physiotherapy program that is automated yet personalized through the ubiquitous and powerful mobile phone offers a promising, scalable solution for the majority of patients, reducing manpower demands while improving access to care.

Bahadori et al [11] emphasize that the development of TKA-related apps often fails to incorporate patient input, which can undermine the effectiveness of these interventions. Given the complexity of rehabilitation, post-TKA recovery typically requires personalized, physiotherapist-guided care [5,6,12]. To improve adherence, this study uses a person-based framework in developing the proposed solution. This framework ensures that behavior change interventions are grounded in a comprehensive understanding of users’ perspectives, experiences, and psychosocial contexts [13]. By incorporating cultural relevance [14] and addressing adherence barriers, the intervention can then be designed to be effective, sustainable, and accessible to diverse populations.

Our primary objective is to identify patient needs, behaviors, and preferences to inform the development of a user-friendly and effective personalized automated smartphone telemonitored app that supports recovery post-TKA.

## Methods

### Study Design

We conducted a qualitative exploration using a person-based approach [13] to inform the development of a digital health behavior change intervention. To ensure methodological rigor, we adhered to the COREQ (Consolidated Criteria for Reporting Qualitative Research), which guided our assessment of the research team, study methods, findings, analysis, and interpretations [15]. Our data collection involved individual in-depth interviews with physiotherapists who work with patients with TKA, as well as patients who had undergone a unilateral TKA within the past 6 months. These participants were selected for their insight into user needs and preferences during TKA rehabilitation.

At the outset of each interview, the interviewer clearly articulated the objectives of the personalized automated smartphone app under development and provided an overview of the various phases of rehabilitation, including preoperation and inpatient and outpatient stages. Given that Singapore General Hospital performs more than half of the nation’s TKA, with more than 2000 surgeries done annually [16], we have established an evidence-based rehabilitation protocol and accompanying information sheet guide [17]. These resources will serve as the foundational elements for the proposed program, ensuring that it is grounded in best practices and tailored to meet the specific needs of patients with TKA.

A semistructured interview guide was developed and piloted for the purpose of this study, organized into two main segments: (1) the contents and functions required by the patient and the physiotherapist for a personalized automated telemonitored rehabilitation app, and (2) the design elements of the user interface to address their specific needs. The questions were informed by the Technology Acceptance Model [18] and the Mobile App Rating Scale assessment [19] covering key interfaces for the recovery and rehabilitation journey, including the menu, dashboard, education materials, exercise module, and assessment tool. By aligning our questions with these domains, we aimed to gain a deeper understanding of user needs. Thus, the study aims to integrate end user insights during co-design to ensure the app’s perceived usefulness, ease of use, and appeal. In line with the person-based approach [13], the interviews also used more open-ended questions and allowed for probing and clarification of responses, to capture deeper insights and provide participants the opportunity to express their perspectives freely.

### Recruitment of Patients

We used purposive sampling to achieve a wide range of patient characteristics (age, gender, socioeconomic status, and education level) to obtain a rich data collection. The study team identified eligible patients through physiotherapists who attend to patients with TKA. Adult patients with previous primary TKA done in Singapore General Hospital, sufficient linguistic capacities to participate in a 1-hour interview, and the ability to navigate at least 1 smartphone app were included. The selection of patients was based on the judgment of the recruiting therapists.

### Recruitment of Physiotherapists

The team contacted physiotherapists from Singapore General Hospital by email or phone. To be eligible for inclusion, physiotherapists needed to have sufficient experience in managing patients undergoing TKA, which was defined as having treated at least 5 of these patients weekly in the past three months. Again, a wide range of physiotherapist characteristics (age, experience, professional qualification, and work setting) was sought to obtain a rich data collection.

### Collection and Analysis of Data

All interviews were conducted by a trained interviewer over Zoom (Zoom Video Communications Inc). The trained interviewer was not part of the study team and had been employed specifically to conduct the interviews, so there was no relationship with participants prior to the interviews. They had a Master of Arts in Sociology, worked as a research associate at the time of the study, and had prior experience conducting interviews for 20 studies. The interviewer was supportive of the idea of a personalized automated telemonitored rehabilitation smartphone app but had no previous work in this area.

The interviews lasted approximately 1 hour and were digitally audio-taped and subsequently transcribed by the same interviewer. In addition, field notes were taken by the team during the interview describing the context of the interview. Apart from relevant study team members, the interviewer, and the participants, no one else was present during the interviews. After each interview had been transcribed, the interviewer used data analysis to check which topics emerged and recruited additional patients and physiotherapists until data saturation was achieved.

The data from the interviews were analyzed using NVivo (version 12.0; QRS International). Thematic analysis was used to identify emerging themes. The analysis was data-driven; the 2 reviewers (ESC and SSK) independently read all transcripts to familiarize themselves with the data and recorded their initial codes. Each reviewer then organized and combined recurrent codes into broader themes. Data triangulation was achieved by cross-checking data sources and involving 3 reviewers (ESC, SSK, and AES) in the study to discuss discrepancies for a consensus. Finally, the main themes were identified based on the coded data in the NVivo. The field notes, transcripts, and themes were intentionally withheld from participants to minimize the potential for bias in their interpretation of their own responses. In the upcoming phase of rapid prototyping, the initial prototype will be presented to the same group of participants, who will then offer feedback on both its content and design.

### Ethical Considerations

This study was approved by the SingHealth Centralized Institutional Review Board (2022/2177, Singapore) and all participants provided written informed consent. Ethical guidelines were strictly followed, with data deidentification ensuring participant privacy during interviews. Participants received a reimbursement of SGD $50 (US $36.98) per session for their time and inconvenience.

## Results

### Participant Characteristics

A total of 11 eligible patients and 4 eligible physiotherapists were invited. A total of 6 (55%) patients and 4 (100%) physiotherapists consented to take part in the study. Reasons for refusal included being busy with other commitments (n=3, 60%) and having no interest to participate in research activity (n=2, 40%). Recruitment for the interviews was done until data saturation was achieved. Among the patients, the median age was 65 (IQR 64-71) years, 50% (n=3) of the participants were male, 83% (n=5) were Chinese, and 67% (n=4) were employed at the point of recruitment. The median age for physiotherapists was 27 (IQR 24‐36) years, and 50% (n=2) had more than 5 years of experience (Table 1).

**Table 1.** Characteristics of patients and physiotherapists participating in the interviews.

Variables	Value
All physiotherapists (n=4)	
Age (years)	
Median (IQR)	24-36
Mean (SD)	39 (16)
Sex (female), n (%)	4 (100)
Education level, n (%)	
Undergraduate degree	2 (50)
Postgraduate degree	2 (50)
PT^a^ experience (years), n (%)	
1‐5	2 (50)
5‐10	1 (25)
>10	1 (25)
TKA^b^ experience (years), n (%)	
<5	2 (50)
5‐10	2 (50)
All patients (n=6)	
Age (years)	
Median (IQR)	64-71
Mean (SD)	70 (7)
Sex, n (%)	
Male	3 (50)
Female	3 (50)
Ethnic group, n (%)	
Chinese	5 (83)
Malay	0 (0)
Indian	1 (17)
Marital status, n (%)	
Married	6 (100)
Number of children, n (%)	
1	2 (33)
2	1 (17)
3	3 (50)
Highest education level, n (%)	
Primary	1 (17)
Secondary	2 (33)
Tertiary	3 (50)
Employment status, n (%)	
Employed	4 (67)
Unemployed	1 (17)
Retired	1 (17)
Housing ownership, n (%)	
Purchased	6 (100)
House type, n (%)	
Public housing flats 3/4/5-rooms	3 (50)
Private apartment or condominium	2 (33)
Landed property	1 (17)
Living arrangement, n (%)	
Living with spouse	3 (50)
Living with spouse and children	3 (50)
Household income (SGD $)^c^, n (%)	
<$ 2000	0 (0)
$2000-$4000	2 (33)
>$4000	3 (50)
Not shown	1 (17)
ADL^d^ limitation, n (%)	
Yes	0 (0)
No	6 (100)
iADL^e^ limitation, n (%)	
Yes (>2)	1 (17)
No	5 (83)
Presence of formal caregiver, n (%)	
Yes	0 (0)
No	6 (100)
Comorbidity, n (%)	
Hypertension	4 (67)
Dyslipidemia	2 (33)
Ischemia heart disease	1 (17)
Asthma	1 (17)
Use of mobile app, n (%)	
WhatsApp	5 (83)
HealthBuddy	1 (17)

a PT: Physiotherapist

b TKA: total knee arthroplasty.

c All income is reported in Singapore dollars (SGD $1=US $0.74).

d ADL: activities of daily living.

e iADL: instrumental activities of daily living.

**Figure 1.**
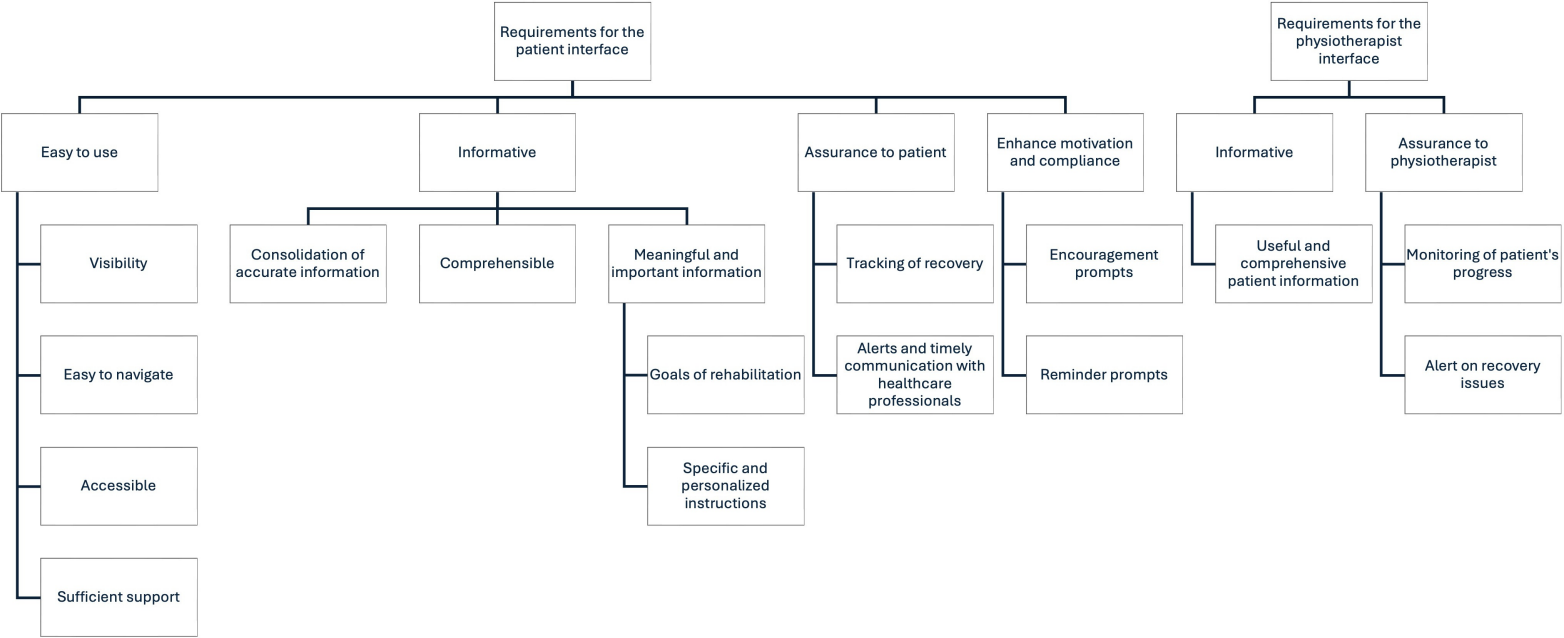
Coding tree.

We conducted 10 individual interviews, which yielded important themes on user requirements for the interface, content, and function of the smartphone app. The coding tree is presented in Figure 1. All participants liked the idea of a telemonitored rehabilitation smartphone app and appreciated the value it would bring:

*Better to do this, don’t need to come down. I come down and do, taxi fare another thing. Then I have to wait ... Might as well I do at home.*

[Patient #5]

*Is good for us. More convenient ... I stay in Tampines, so far. I take 1 hour to and fro ... help us when we are sick, we cannot come down.*

[Patient #6]

### Patient App

#### Easy to Use

As the patients were older than 60 years of age and may not be technologically proficient, there was a unanimous consensus among participants that the app should be easy to navigate and user-friendly. This involves implementing large, visible buttons adorned with comprehensible, prominent headings and fonts. The collective emphasis was on the necessity of organizing information in a systematic manner, with intuitive categorization of information facilitating seamless navigation for enhanced accessibility:

*Honestly, I think the interface needs to be as simple as possible. It shouldn’t be too cluttered, not too many words or images.*

[Patient #4]

Prior to undergoing surgery, patients are inundated with a plethora of information and training materials. Several individuals proposed the integration of a search function to streamline the identification of materials aligning with their specific interests. Additionally, they underscored the significance of information offering customization options to cater to diverse language preferences and auditory needs. Some participants also suggested adding a voice recording feature for those who may have difficulty typing. Participants expressed a consensus on avoiding additional equipment or wearables due to the associated hassles in setup, maintenance, and frequent connectivity issues:

*Search function would definitely help so that not everybody need to start from beginning.*

[Patient #2]

*Machine ... wear and tear ... connection also loosen ... if you can put all these in the app, it would be much better.*

[Patient #1]

Participants recommended that education, whether before surgery or during hospitalization, should involve a personal touch, with an instructor guiding them through the app to ensure comprehension and compliance. Following this initial human interaction, the app can seamlessly take over. Additionally, participants stressed the crucial need for readily available tech support, citing past experiences with apps that malfunctioned and lacked prompt assistance:

*For those not so well-versed, they need at least 1 or 2 sessions hands on so they can be more confident to do it on their own.*

[Patient #1]

#### Informative

Patients highlighted challenges with the untimely delivery of information, leading to either overwhelming or insufficient details. The current mode of patient education, conveyed through booklets, often proves impractical as it is prone to misplacement, lacks bite-sized segments, and lacks a searchable format for specific queries. Participants expressed a desire for accurate, condition-specific information available in a timely manner, encompassing both pre- and postsurgery aspects. Additionally, they emphasized the importance of having clear goals and expectations. By delivering the right information at the right time, the mobile app can empower patients to make informed decisions, better anticipate and understand the recovery process, and actively participate in their recovery journey. Participants also sought insights and guidance on when and how to access professional support:

*I tried to search on Google ... so the information may not be correct ... it may be something else ... It is good to give information of what will be faced after surgery.*

[Patient #3]

The information should be comprehensive, easily understandable, free from technical jargon, and presented succinctly. Recognizing the need for repeated reference, participants see the advantage of housing these materials in an app for convenient access:

*You want to give the patient information that he needs, you want to avoid giving him information that he doesn’t need and devalue the app.*

[Patient #2]

*Some after surgery they call in to a physio, not every time the physios are available. But if you see from the app, you can always play it again and again until they understand.*

[Patient #2]

Among the requested content, there was a distinct emphasis on the imperative need for comprehensive information concerning pain management and swelling strategies, deemed vital for ensuring a successful recovery.

Guidance for pre and postsurgery exercises can be effectively conveyed through a combination of images and videos, tailored to individual needs and abilities. The instructions should encompass precise targets and goals, providing clarity on the criteria for discharge from physiotherapy:

*Pictures and videos people can follow ... cannot have a lot of words.*

[Patient #5]

*How would the patient know when they can actually stop (physiotherapy) or come to a point where there is a full recovery.*

[Patient #4]

#### Assurance to Patient

Just as in a physical outpatient physiotherapy session, patients emphasized the importance of reporting their progress for tracking and monitoring, seeking assurance in the process:

*To see that you are improving is important.*

[Patient #3]

They expressed a desire to gain insights into whether they were on the correct path to recovery and to understand their performance relative to others. Additionally, they emphasized the crucial need for feedback on the accuracy of their rehabilitation efforts and prompt notifications if their recovery deviated from the expected trajectory. While patients acknowledged the positive impact of comparing themselves to the same age group and gender for recovery metrics—providing a sense of accomplishment and motivation—they also recognized the potential downside. They noted that for those not progressing well, such comparisons might lead to demoralization.

Understandably, patients expressed the importance of having access to health care professionals for addressing any concerns:

*Some patients who are already very worried and verbalized that they want to have a session with a physiotherapist or a doctor to check on them, we can arrange that.*

[Patient #2]

They emphasized that while the app is a valuable tool, it cannot entirely replace the role of physiotherapists. The consensus was that the app should complement physiotherapy sessions. Patients speculated that this perspective might evolve as society becomes more open, particularly with the younger generation aging.

In a majority of responses, patients individually conveyed a preference for at least 1 to 2 physiotherapy sessions, citing that the app does not provide them with 100% assurance. Many older adults expressed discomfort with the lack of human interaction, highlighting the enduring significance of personal engagement in health care:

*Sometimes we are not very sure are we doing it correctly or not, but if we go for physio, we can understand better.*

[Patient #6]

#### Enhance Motivation and Compliance

Participants recommended incorporating functions to boost motivation and adherence to rehabilitation. They highlighted the utility of features such as tracking sessions with an exercise log. Setting alarms and receiving prompts to complete exercises, coupled with the ability to monitor personal progress, were identified as key elements that would significantly enhance motivation and compliance. In addition, the app should incorporate features for positive reinforcement and encouragement upon completion, further enhancing the user experience and motivation for rehabilitation:

*At the end over every exercise, every set that I do, the app actually congratulates me, so it’s motivating. It will say well done you’ve completed this function or this set. That helps. Although you know it’s just automatic, but it give you a bit of a boost, like oh I finished something!*

[Patient #4]

*The reward part is quite individual. For me, I’m quite concerned for my own health and I don’t need any monetary rewards. But if the application can tell me well done, you have achieved your target, to me it is good enough.*

[Patient #1]

### Physiotherapist App

#### Informative

Physiotherapists expressed the need to have easily accessible patient information within the program to facilitate seamless follow-up coordination. They emphasized the efficiency of receiving prompts when patients are not progressing well, as it would save valuable time for the clinicians. Streamlining this information and notification process was considered essential to enhance the effectiveness of patient care and monitoring:

*It will be good to keep a lookout on their range, like how much they can extend the knee … how much they are walking on a daily basis, what is their pain score when doing the exercises…so that we can see that they are improving.*

[Physiotherapist #3]

#### Assurance to Physiotherapist

Several reporting parameters, encompassing both subjective and objective outcomes such as pain levels, range of motion, walking ability, and patient-reported outcome measures, were identified as crucial. These parameters serve as essential indicators to reassure physiotherapists about the well-being and progress of their patients. The inclusion of these comprehensive metrics allows for a more thorough and accurate assessment of patient outcomes, contributing to the overall effectiveness of the rehabilitation program:

*For physios we look quite holistically… so we will see how they are as well, walking at home and all. So I’d prefer to have other outcome measures and patients subjectively how they feel, so that we can see the treatment is really working.*

[Physiotherapist #3]

## Discussion

### Principal Results

Patients outlined requirements for ease of use, the provision of information and reassurance, and the enhancement of motivation and compliance. Physiotherapists raised themes for the provision of information and reassurance. Beyond requirements, participants anticipated several benefits of the app. These included improved access to patients, heightened motivation for rehabilitation, enhanced convenience, and potential cost savings. Participants shared the collective vision for the app to not only meet specific needs but also to create a positive impact on patient engagement, overall motivation, and the efficiency of health care delivery.

### Strengths and Limitations

To our knowledge, this is the first study to use a person-based framework to systematically investigate the requirements for an automated, smartphone-based solution tailored to the rehabilitation needs of patients post-TKA. By addressing a significant gap in current rehabilitation practices, our findings emphasize the potential of integrating digital health technologies into personalized care. A key feature identified by both patients and physiotherapists is the need for outcome tracking, real-time monitoring, and a feedback loop to facilitate the personalization and automation of care delivery. These features not only enhance patient motivation but also provide reassurance to both patients and clinicians, promoting improved adherence and engagement throughout the rehabilitation process.

The inclusion of users during the design phase yielded crucial revelations, pinpointing significant content and features essential for an automated telemonitored smartphone app. This approach is pivotal not only for ensuring widespread adoption but also for enhancing patient adherence and ultimately elevating overall patient outcomes. Further studies and app developers should, however, continue to involve patients in an iterative prototyping and development phase including the use of think-aloud interviews [13] to receive specific feedback for the actual app.

As the participants in this study may not fully represent the entirety of patients undergoing TKA, there may be limitations in generalizability. However, the characteristics of our participants, as evident in their diverse age range, gender distribution, socioeconomic status, and educational levels, contribute to a broad spectrum of perspectives in our data collection.

Despite the relatively small sample size, data saturation was achieved during discussions. Notably, studies, such as those referenced by Nielsen and Laudauer [20], propose that a sample size as modest as 5 users can uncover a significant percentage (75%‐80%) of usability issues in the design of user-centered systems. Furthermore, empirical data studies suggest that saturation can be reached within a narrow range of interviews, especially in studies with relatively homogenous populations and narrowly defined objectives [21], aligning with the characteristics of this study.

### Comparison With Prior Work

The existing literature on telerehabilitation, including in post-TKA cases [22,23], closely aligns with the findings related to both patient and physiotherapist apps.

#### Easy to Use

Our findings highlight the importance of user-friendliness and accessibility for older adults, a need that is strongly supported by various studies advocating for intuitive telerehabilitation designs [24]. User-friendly interfaces that cater to the technological abilities of older users are essential, with features like large, clear buttons, and well-organized information can significantly enhance usability [25,26]. This echoes participants’ feedback which stressed the need for simplicity and clarity in navigation.

Presurgery, patients often face information overload. The ability to quickly locate relevant materials can enhance their experience [27]. Hughes et al [28] emphasize the crucial role of accessible patient information in improving the effectiveness of rehabilitation programs, aligning with the feedback from our participants.

#### Informative

Participants expressed a strong preference for comprehensive, easily understandable information devoid of technical jargon which is consistent with research emphasizing clear communication in health care apps [24]. The participants’ request for visual aids, such as images and videos for exercise guidance, is supported by studies showing that these formats can enhance understanding and information retention [29].

Additionally, the need for specific content on pain management and recovery strategies was frequently highlighted by participants and is well-documented in studies that stress the importance of addressing these areas for successful rehabilitation outcomes [30-33]. Aligned with existing literature [29,32], participants consistently expressed the desire for timely, accurate, condition-specific information, empowering them to make informed decisions about their recovery.

#### Assurance to Patient

The use of a feedback loop, where patients receive notifications about their performance and recovery progress, is crucial for maintaining engagement and ensuring that patients feel supported throughout their rehabilitation journey. This is particularly important for older adults, who often require extra reassurance and motivation to stay compliant with their rehabilitation exercises [33,34].

Additionally, both the findings and literature emphasize the importance of a personal touch in education and support. Participants expressed the importance of initial guidance from health care professionals to build confidence in using the app. This sentiment is echoed in the literature, which highlights clinician-patient communication as a critical factor in fostering a supportive environment [33,35]. While telerehabilitation apps provide valuable resources, they should be viewed as complementary to direct interactions with health care professionals rather than as a replacement [36].

#### Enhance Motivation and Compliance

Our findings align with the recommendations of Mrklas et al [32] and Bahadori et al [11], who advocate for the inclusion of self-tracking features in mobile apps to enhance patient engagement and ensure that the information provided is both robust and contextualized. Research further shows that including motivational elements, such as exercise logs, reminders, and congratulatory messages, can significantly improve patient adherence to rehabilitation programs [29,33,37]. However, it is noteworthy that participants did not specifically mention gamification elements, which have been shown to aid compliance in telerehabilitation [33]. This suggests that gamification may not be a priority for this particular group of patients.

### Implication for Future Research

The insights generated from this study have identified essential design elements, focusing on patient preferences and adherence barriers, which are guiding the development of a personalized, automated smartphone app for post-TKA rehabilitation. This app is currently being evaluated in a randomized controlled trial (ClinicalTrials.gov: NCT06248034). Findings from this trial will provide valuable evidence on the effectiveness of digital health interventions in improving post-TKA outcomes. Additionally, this research defines the foundational requirements for creating scalable, patient-centered digital solutions that reduce manpower demands and can be adapted to diverse rehabilitation contexts, contributing significantly to the advancement of digital health and personalized care.

In summary, the findings from the patient and physiotherapist apps align closely with existing literature on telerehabilitation. The emphasis on user-friendly design, timely information delivery, motivational features, and the importance of clinician support are all well-supported by current research and correspond with models of learning such as the Illeris model [38], which incorporates all 3 dimensions of learning: content, incentive, and interaction. A key feature identified by both patients and physiotherapists is the need for outcome tracking, real-time monitoring, and a feedback loop to facilitate the personalization and automation of care delivery. These features not only enhance patient motivation but also provide reassurance to both patients and clinicians, promoting improved adherence and engagement throughout the rehabilitation process.

### Conclusions

This study provides a comprehensive exploration of the perspective of both patients and physiotherapists regarding the requirements for a personalized automated smartphone app designed for telemonitored rehabilitation following TKA relevant to an Asian context. To ensure the program is both automated and tailored to each individual, the findings reveal a strong consensus from both groups on the need for automating rehabilitation progression and outcome monitoring.

Such an app could serve as a powerful catalyst for boosting patient motivation, satisfaction, and active engagement throughout the rehabilitation process. By providing real-time feedback and progress tracking, it would empower patients to take ownership of their recovery, fostering a sense of agency and commitment to their rehabilitation goals. Furthermore, the app would equip health care providers with tools to identify patients who may be deviating from their expected recovery trajectory, enabling timely, targeted interventions for those in need of professional guidance. This would ultimately optimize patient outcomes and enhance the overall effectiveness of rehabilitation programs.

The insights from this study provide clear guidelines for developing a user-centric smartphone app that meets the needs of both patients and health care providers in this region.

## Abbreviations

COREQ: Consolidated Criteria for Reporting Qualitative Research
TKA: total knee arthroplasty
